# Evidence of Reduced Virulence and Increased Colonization Among Pneumococcal Isolates of Serotype 3 Clade II Lineage in Mice

**DOI:** 10.1093/infdis/jiae038

**Published:** 2024-01-29

**Authors:** Ognjen Sekulovic, Caitlyn Gallagher, Jonathan Lee, Li Hao, Stavros Zinonos, Charles Y Tan, Annaliesa Anderson, Isis Kanevsky

**Affiliations:** Pfizer Inc, Bacterial Vaccines and Technology, Pearl River, New York; Pfizer Inc, Bacterial Vaccines and Technology, Pearl River, New York; Pfizer Inc, Bacterial Vaccines and Technology, Pearl River, New York; Pfizer Inc, Bacterial Vaccines and Technology, Pearl River, New York; Pfizer Inc, Bacterial Vaccines and Technology, Pearl River, New York; Pfizer Inc, Early Clinical Development, Collegeville, Pennsylvania; Pfizer Inc, Bacterial Vaccines and Technology, Pearl River, New York; Pfizer Inc, Bacterial Vaccines and Technology, Pearl River, New York

**Keywords:** *Streptococcus pneumoniae*, serotype 3, virulence, carriage, clade II

## Abstract

Recent phylogenetic profiling of pneumococcal serotype 3 (Pn3) isolates revealed a dynamic interplay among major lineages with the emergence and global spread of a variant termed clade II. The cause of Pn3 clade II dissemination along with epidemiological and clinical ramifications are currently unknown. Here, we sought to explore biological characteristics of dominant Pn3 clades in a mouse model of pneumococcal invasive disease and carriage. Carriage and virulence potential were strain dependent with marked differences among clades. We found that clinical isolates from Pn3 clade II are less virulent and less invasive in mice compared to clade I isolates. We also observed that clade II isolates are carried for longer and at higher bacterial densities in mice compared to clade I isolates. Taken together, our data suggest that the epidemiological success of Pn3 clade II could be related to alterations in the pathogen's ability to cause invasive disease and to establish a robust carriage episode.

## Teaser

Pneumococcal serotype 3 clinical isolates display variable and clade-dependent biological characteristics in a mouse model of pneumococcal invasive disease and carriage.

*Streptococcus pneumoniae* (pneumococcus) maintains a complex relationship with its human host. It mainly resides as a harmless commensal of the upper respiratory tract but can also invade essentially sterile sites, causing invasive disease [1]. While the trade-off between chronic and acute infection is a fundamental adaptive challenge many pathogens face, pneumococcal genetic and capsular diversity allows for stable coexistence of lineages that considerably vary in their pathogenic ability [2]. In addition, strong selective pressures such as vaccine and antibiotic use shape pneumococcal populations on genotypic and phenotypic levels. Whole-genome phylogeny was recently applied to examine population-scale genetics of serotype 3 pneumococci (Pn3) [3–5]. A spatiotemporal shift among major lineages was described, including global dissemination of a variant termed clade II. The cause of the clade II emergence is unclear with no evidence of vaccine escape events. While alterations in antigenic, competence, and antimicrobial resistance profiles are hypothesized as likely contributors, experimental confirmation linking genomic and phenotypic features of clade II to its recent emergence is still lacking.

In this manuscript, we describe phenotypic profiling of a collection of Pn3 isolates associated with distinct clades in relevant mouse models of pneumococcal disease. We find evidence of reduced virulence and low invasiveness but superior carriage potential among clade II isolates relative to clade I isolates. We discuss these findings in the context of the current serotype 3 epidemiology.

## METHODS

### Bacterial Strains and Growth Conditions

*Streptococcus pneumoniae* serotype 3 clinical isolates were used for all experiments (Supplementary Table 1). Isolates were obtained through Pfizer's global Antimicrobial Testing Leadership and Surveillance program (ATLAS) except isolate ATCC 6303, which was sourced from American Type Culture Collection. Isolates were routinely cultured at 37°C with 5% carbon dioxide (CO_2_) on trypticase soy agar plates supplemented with 5% (vol/vol) sheep blood or Todd-Hewitt with 0.5% yeast extract broth (THB) supplemented with 300 U/mL of catalase.

### Preparation of Bacterial Challenge Stocks

Standardized bacterial challenge stocks were prepared by growing pneumococcal isolates on blood agar plates overnight and then inoculating 100 mL of fresh THB + catalase at optical density 600 nm (OD_600_) = 0.01. Bacterial cultures were grown to mid-late exponential phase (OD_600_ = 0.6–0.8) and aliquots stored at −80°C with 10% glycerol. Bacterial challenge doses were prepared by serial dilutions of 1 vial of frozen challenge stocks in sterile phosphate-buffered saline (PBS), pH = 7.4. Target challenge doses ranged between 10^2^ and 10^7^ colony-forming units (CFU)/animal. Challenge doses were confirmed retrospectively by count of viable bacteria following plating on blood agar plates approximately 1 hour after challenge.

### *Streptococcus pneumoniae* Mouse Challenge Model

All procedures performed on animals were in accordance with regulations and established guidelines and were reviewed and approved by an Institutional Animal Care and Use Committee or through an ethical review process. Previously described murine models of pneumonia and carriage were used to characterize the virulence potential of select Pn3 isolates [6–8]. Naive female outbred Swiss–Webster mice (Taconic Laboratories), 13–15 weeks of age, were lightly anesthetized by isoflurane inhalation and inoculated intranasally with either PBS or bacterial suspension. For the invasive challenge model, inoculum was given in a volume of 0.04 mL in a single nostril, which ensures optimal delivery of bacteria to the lungs with minimal sample loss. For the nasopharyngeal carriage model, inoculum was given in a volume of 0.01 mL split equally in both nostrils to ensure bacteria are reaching both nasal passages. Mice were closely monitored by trained personnel for the entire duration of study. A clinical score guide was used to record clinical signs, evaluate disease progression, and determine eventual experimental endpoint. For carriage endpoint studies, mice were euthanized via CO_2_ inhalation at either day 14 or day 21 postchallenge. After euthanasia, the nasopharynx was flushed with 0.5 mL of sterile PBS by retrograde nasal lavage through the exposed trachea. Bacterial loads were determined by plating 0.1 mL of nasal washes on blood agar plates and incubating overnight at 37°C with 5% CO_2_.

### Whole-Genome Sequencing and Phylogenetic Analysis

Genomic DNA from Pn3 pneumococcal isolates was extracted and then sequenced on the Illumina MiSeq, with 2 × 250 bp paired-end sequencing chemistry. De novo assembly of the genome was performed using the CLC Genomic Workbench (v20) with default settings.

Whole-genome core alignments and phylogenetic analysis were performed using an in-house developed application (miTree v2.0, unpublished). The pipeline integrates Parsnp [9], a rapid genome aligner from Harvest suite, with customized phylogenetic tree visualization tool. To identify Pn3 clades, representative isolates of each clade were selected from previous publication [3] and combined with our ATLAS Pn3 isolate collection. Global Pneumococcal Sequence Cluster (GPSC) was determined using PopPunk v2.6.1 [10, 11]. Amino acid conservation of prevailing pneumococcal protein antigens was profiled using the large-scale blast score ratio (LS-BSR) pipeline using the tblastn function [12]. In-frame nucleotide sequence of pneumococcal antigens used as query is included in the Supplementary Materials. This study was registered at the National Center for Biotechnology Information under BioProject accession number PRJNA996189.

### Statistical Analysis

Statistical analysis was done using R software v4.1.0 [13]. Differences in the bacterial CFU/mL levels among major clades I (I-α and I-β pooled) and II were evaluated using analysis of variance on ranks. Differences in the proportion of responses below the limit of detection among clades were analyzed using a generalized mixed effects model with a binomial link. A bootstrap method was used to assess the power of detecting differences among the clades.

## RESULTS

### Pn3 Clade II Isolates Display Lower Virulence Potential in Mice

Previous studies reported limited evidence of in vitro phenotypic differences among isolates from distinct Pn3 phylogenetic clades [3, 5]. We reasoned that a mouse model of pneumococcal invasive disease might capture more accurately potential differences of relevant in vivo phenotypes. Therefore, we evaluated the virulence potential of select Pn3 isolates in naive mice. Virulence was defined as the relative capacity of an isolate to cause damage (ie, disease) in mice leading to decrease in survival over a period of 14 days and across a range of challenge doses [14]. Representative pneumococcal strains from clade I and clade II were chosen to maximize the diversity in geographical and temporal sites of isolation (Supplementary Table 1). All tested isolates belong to the multilocus sequence type 180 (ST180) except 2 clade I-β isolates that belong to the ST505. ST180 and ST505 differ by only 2 typing loci and likely share similar genetic background, hence their inclusion in the phylogenetic and phenotypic assessment [5]. All isolates belong to GPSC 12. For reference purposes, we also included a serotype 3 strain ATCC 6303 that belongs to a different multilocus sequence type (ST378).

**Figure 1.**
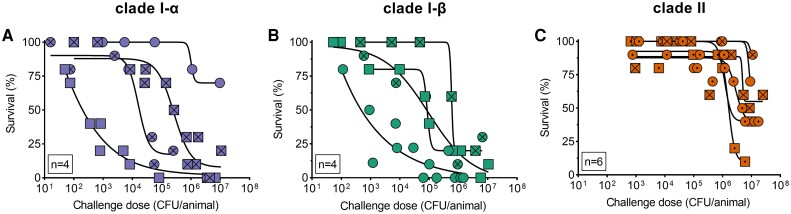
Pneumococcal serotype 3 (Pn3) clade II isolates require higher challenge doses to reach study endpoint of euthanasia among challenged mice. Survival among challenged mice was evaluated for pneumococcal isolates from clade I-α (n = 4, *A*), clade I-β (n = 4, *B*), and clade II (n = 6, *C*). Survival as a function of challenge dose was modeled using 4-parameter logistic (4PL) dose-response. Each data point represents survival percentage from a group of 10 mice. A minimum of 5 challenge doses covering ≥10 000-fold range in colony-forming units (CFU)/animal was used for each tested isolate.

**Figure 2.**
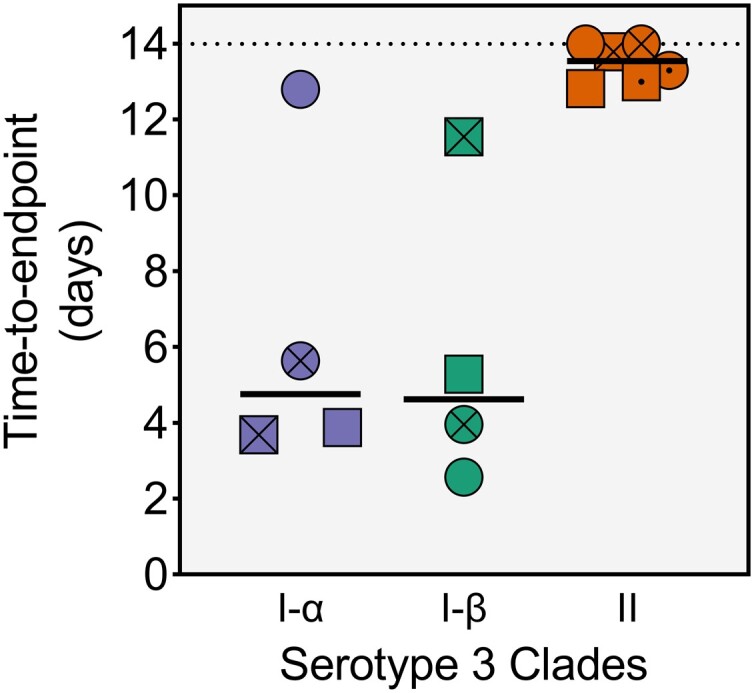
Mice challenged with pneumococcal serotype 3 clade II isolates survive for longer periods. Average time-to-endpoint (euthanasia) occurrence was recorded following challenge of a group of 10 mice with 1 × 10^6^ colony-forming units/animal of each isolate. Animals surviving the 2-week period were sacrificed and time to death was recorded as 14 days. Horizontal line represents the median and the dotted line represents the study duration.

As expected, an increase in challenge doses is typically reflected in reduced survival among challenged mice for all tested isolates (Figure 1). The rate at which the mortality occurred with increasing challenge doses varied by isolate and reflected virulence potential, commonly measured by the median lethal dose 50% (LD_50_) parameter [15]. The variability in virulence was more pronounced among isolates from clade I-α with the LD_50_ values ranging from 2.15 × 10^2^ CFU/animal to 1.93 × 10^5^ CFU/animal. One isolate did not reach 50% mortality threshold at the maximal tested challenge dose of 9.60 × 10^6^ CFU/animal. Isolates from clade I-β displayed similar variability in virulence with LD_50_ ranging from 5.27 × 10^2^ to 7.23 × 10^5^ CFU/animal. In contrast, isolates from clade II displayed low and uniform virulence profiles with LD_50_ values consistently above 1 × 10^6^ CFU/animal. In addition, 3 clade II isolates did not reach the 50% mortality threshold at the maximal challenged doses of ≥9.00 × 10^6^ CFU/animal. Time-to-endpoint analysis at a fixed dose of 1 × 10^6^ CFU/animal also illustrates the lower disease potential of clade II strains (Figure 2). Nevertheless, these isolates are not considered avirulent since they caused 10%–40% mortality at the highest challenge doses.

A reference serotype 3 strain, ATCC 6303, also caused dose-dependent mortality among challenged mice with a virulence profile characterized by an LD_50_ of 4.86 × 10^5^ CFU/animal (Supplementary Figure 1*A*). This is generally in line with other published studies [16–19]. Discordance in lethality outcomes can be attributed to differences in mouse species, age, challenge route, and inoculum size.

Taken together, phenotypic profiling in a mouse model of invasive pneumococcal disease suggests that Pn3 clade II isolates cause, on average, less mortality and hence could be less virulent compared to isolates from clades I-α and I-β. We next sought to explore the capacity of the same set of isolates to successfully colonize the murine nasopharynx in a nonlethal mouse model of pneumococcal carriage.

### Pn3 Clade II Isolates Are Carried for Longer and at Higher Rates in Mice

Pneumococcal carriage, defined as the ability of an isolate to establish a temporary balance with the host leading to extended persistence on mucosal tissues, is an essential aspect of pneumococcal life cycle [20]. Having observed that Pn3 clade II isolates induce lower virulence in mice, we sought to determine the capacity of the same set of Pn3 isolates to persist in the nasopharynx of naive mice.

**Figure 3.**
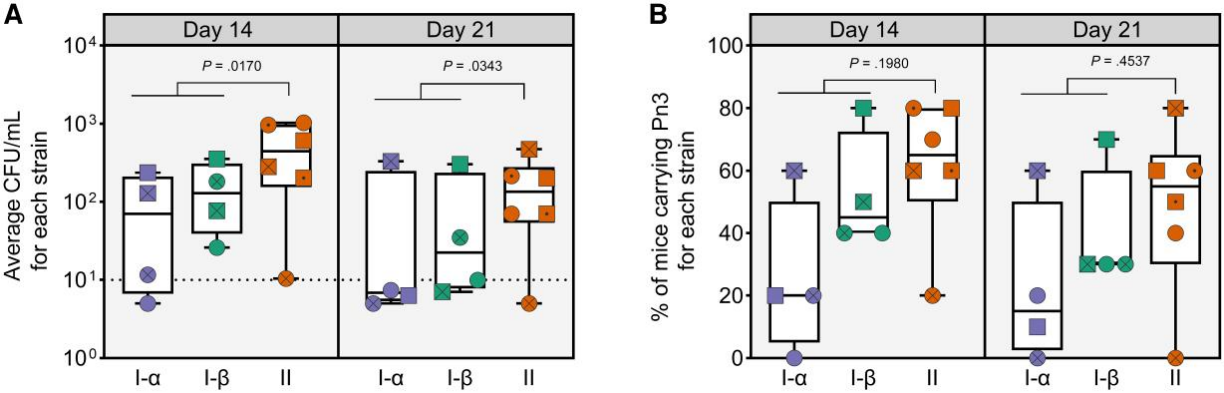
Pneumococcal serotype 3 (Pn3) clade II isolates are carried for longer and at higher levels in the nasopharynx of challenged mice. Each data point represents the geometric average bacterial load (*A*) or percentage of colonized animals (*B*) from a group of 10 mice, challenged with 10^4^ colony-forming units (CFU)/animal with a single Pn3 isolate. Within a clade, distinct isolates are marked with unique symbols and the assignment is kept consistent for day 14 and day 21 panels. Horizontal dotted line (*A*) indicates the limit of detection (LOD) of 10 CFU/mL. If no viable pneumococci were detected, half-LOD (5 CFU/mL) was reported. An animal was considered colonized (*B*) when at least 1 pneumococcal colony was detected following plating of 0.1 mL of nasal wash samples, corresponding to bacterial counts ≥10 CFU/mL.

Bacterial burden in the nasopharynx was quantified by plating nasal washes collected following euthanasia at 14 and 21 days postchallenge. Distinct isolates, regardless of the clade, were carried at different densities in the nasopharynx as illustrated by the geometric average CFU/mL levels for individual isolates (Figure 3*A*). Carriage density was generally higher at day 14 compared to day 21 with clade I-α and I-β exhibiting signs of accelerated clearance. We observed low pneumococcal density in nasopharynx in most animals on day 21 for 3 of 4 isolates from clade I-α and 2 of 4 isolates from clade I-β. In contrast, 5 of 6 isolates from clade II displayed relatively high bacterial density at day 21. A single isolate from clade II failed to establish a successful carriage episode on both days, likely indicating a complete impairment of long-term carriage capacity. Similarly, the percentage of mice with detectable levels of pneumococci in the nasopharynx was generally greater at day 14 compared to day 21 for all clades (Figure 3*B*). At day 21, 40%–80% of mice had measurable levels of pneumococci for 5 of 6 clade II strains while only 1 of 4 isolates from each of clade I-α and clade I-β had >30% carriage rate. In comparison, ATCC 6303 isolate displayed relatively stable carriage levels over time with 70% and 60% of animals carrying pneumococci at 14 and 21 days postchallenge, respectively (Supplementary Figure 1*B*).

We focused our statistical analysis on the differences among the 2 major clades (I and II) without controlling for the subclades. We confirmed that overall pneumococcal density in nasal washes in mice is statistically higher following challenge with isolates from clade II versus clade I at both timepoints (Figure 3*A*). Statistical comparisons were affected, in part, by the low number of tested isolates, resulting in relatively low statistical power of 0.77 for the clade comparison on day 14, and 0.60 on day 21. Differences among the percentage of colonized mice at both timepoints were not statistically significant (Figure 3*B*); however, the absence of meaningful statistical power (0.16 at day 14 and 0.02 at day 21) preclude an accurate analysis of this dataset. Taken together, the data suggest that clade II isolates have a greater ability to establish and maintain carriage than clade I isolates.

### Variation Among Common Protein Antigens Does Not Correlate With Phenotypic Differences

**Figure 4.**
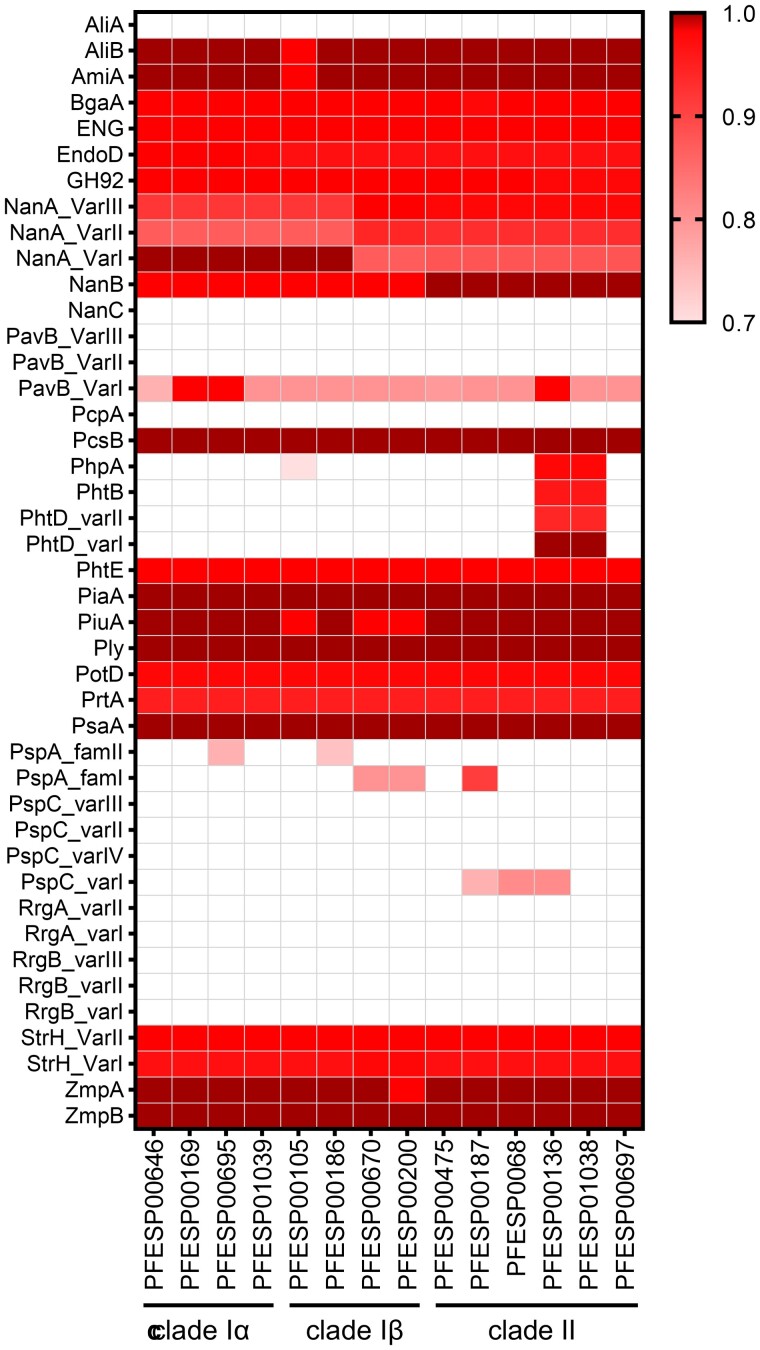
Prevailing pneumococcal protein antigens show sparse distribution among select serotype 3 isolates. Heatmap representing amino acid conservation of prevailing pneumococcal protein antigens in select serotype 3 isolates. Antigen homologues were probed against serotype 3 genomes using large-scale blast score ratio (LS-BSR) pipeline and the LS-BSR was used to build the heatmap. Score interpretation: <0.7 (clear cell labels), absent; 0.7–0.99 (gradient cell labels), present with moderate to minor sequence variations; 1 (dark cell labels), present with no sequence variation. Bacterial strains are grouped by clades (bottom) with protein antigens and respective variants in alphabetical order (left).

We evaluated the conservation of 30 prevailing protein antigens and their most common variants using the LS-BSR tool (Figure 4). Five antigens (NanC, AliA, RrgA, RrgB, and PcpA) were absent from all isolates based on the large-scale BLAST score ratio cutoff of 0.7, approximately corresponding to 60% amino acid global sequence identity. Thirteen antigens were present in all isolates (score ratio >0.7) and showed little sequence variation (BgaA, StrH, EndoD, GH92, ENG, PsaA, PotD, PrtA, PhtE, Ply, PiaA, PcsB, and ZmpB). The remaining 12 antigens showed a variable conservation profile and sequence divergence. For example, pneumococcal histidine triad proteins PhpA, PhtB, and PhtD appear absent from all but 2 clade II isolates. Similarly, major surface antigen PspA is present in only 3 isolates (2 clade I-β and 1 clade II), whereas PspC is present only in 2 clade II isolates. Most but not all strains appear to be carrying variant I of the highly variable pneumococcal adherence and virulence factor B (PavB). Neuraminidases NanA and NanB are present in all isolates and display some clade-specific variability.

## DISCUSSION

The global rise of the phylogenetic clade II within the serotype 3 clonal complex 180 (CC180) was ascribed to an alternate antigenic, competence, and antimicrobial resistance profile [3, 5]. These observations were inferred from comparative analysis of genomic sequences with little experimental validation. While genomic analysis can provide important associative links, it offers no information about causal relationships. In this study, we sought to experimentally assess the relevance of Pn3 clade II emergence by leveraging a suitable mouse model of pneumococcal invasive disease and carriage.

Our data indicate that virulence of serotype 3 in mice is strain dependent (Figure 1). For example, clade I isolates displayed relative LD_50_ differences that spanned over 4 orders of magnitude (Supplementary Table 1). While clade II isolates exhibited less variable LD_50_ values, survival rates in mice challenged with high doses varied from 90% to 10%. Phenotypic variability among pneumococcal isolates of the same serotype or genotype has been observed previously, further supporting the notion that genetic factors other than the capsule play a vital role in pneumococcal pathogenesis [21–26]. Therefore, one should avoid overinterpretation and generalization of findings regarding pneumococcal biology based on one or a few isolates in any given context.

Our data also suggest that clade II isolates display a relatively lower virulence profile (Figure 1). While clade II isolates retained their pathogenic potential, higher challenge doses were required to cause substantial mortality in mice compared to clade I isolates. Direct comparison of LD_50_ between clades was unfeasible since several clade II isolates never reached the threshold of 50% mortality. Instead, we performed a time-to-endpoint analysis at a fixed challenge dose and confirmed that mice survive for longer following challenge with clade II compared to clade I isolates (Figure 2). Serotype 3 is known to cause a spectrum of pneumococcal syndromes in people, often with a severe clinical course complicated by empyema, necrotic lung, or occult bacteremia with fatal outcome [27–29]. It is unclear if this association was driven by specific Pn3 lineages such as the historically predominant clade I and if the current clinical course associated with Pn3 infections is changing, potentially in response to the global predominance of the clade II.

Stable association with the host is another crucial aspect of pneumococcal biology. In our mouse model of carriage, clade II isolates were carried for longer and at higher bacterial densities compared to clade I isolates (Figure 3). Isolates from clade I-β appear to have an intermediate phenotype; however, the low sample size precludes firm conclusions. While it is well recognized that nasopharyngeal carriage precedes invasive disease, the temporal relationship between acquisition, persistence, and invasiveness is less clear. Longitudinal studies, reviewed by Simell et al [30], suggest that incidence of invasive disease is associated with the acquisition of new serotypes rather than extended carriage. Whether this constitutes a general feature of pneumococcal biology remains to be determined; however, it might explain why in our study Pn3 clade II isolates establish more robust carriage episodes in mice with less concurrent invasive disease. For example, several clade I isolates displayed significant invasive capacity, demonstrated by their ability to translocate from nasopharynx and cause systemic disease over the course of the study in the nonlethal carriage model, while clade II isolates remained noninvasive (Supplementary Table 1). Once again, this reflects the lack of consensus regarding the invasiveness of serotype 3 in real-world settings [31]. While the seminal study by Brueggemann et al provided early evidence of low invasive potential of Pn3 isolates in children from Oxford [32], a recent study by Colijn et al calculated a relatively elevated invasiveness odds ratio for Pn3 compared to all other serotypes in their meta-analysis of epidemiological datasets [33]. In the meta-analysis from Balsells et al, the invasive disease potential of Pn3 was not significantly different from 19A [34]. Recent epidemiological analysis from healthy children aged <5 years in the community in Blantyre, Malawi, found low invasiveness odds ratio for Pn3 [35]. Taken together, it is conceivable that different phylogenetic lineages of Pn3 could have distinct invasive disease potential, thus further blurring the epidemiological picture of pneumococcal pathogenesis.

The mouse remains the most commonly used animal model for studying pneumococcal disease [6]. In addition, we chose pneumococcal isolates that encompass a range of geographical and temporal sources, thus making it possible to potentially draw broader conclusions about Pn3 biology. As invasive pneumococcal disease is typically preceded by a carriage episode, one would expect carriage and invasive isolates to share the same genotype and similar phenotypes. However, the anatomical site of isolation was previously shown to correlate with virulence in mice, suggesting stable pneumococcal niche adaptations [36]. This could be attributed to bacterial adaptations to environmental factors such as anatomical site-specific carbohydrates [37–39] and alteration in gene expression due to epigenetic [40, 41] or genetic mutations [42, 43]. As most of the isolates used in this study originate from cases of clinical invasive disease, it remains to be seen whether clade I and clade II pneumococci isolated from the nasopharynx (ie, carriage isolates) would reflect similar phenotypic features.

Finally, profiling of common antigens did not identify a clear correlation with observed in vivo phenotypes. Only neuraminidases NanA and NanB display some level of clade-specific association, while other antigens are either absent or present sporadically. Differences in methodologies likely explain observed discrepancies in conservation of select antigen such as PspA and PspC between our study and previously published reports [3, 5]. For example, we based our analysis on amino acid sequence comparison using blast score ratios with user-defined threshold cutoffs [12], whereas previous reports exploited mapping of raw genomic sequencing reads to an antigen variant database. In addition, high sequence variability could also explain why certain antigens appear absent from select isolates (eg, PspA, PspC, PavB). Last, experimental validation is required to determine whether sequence variation of neuraminidases, other antigens of core or pan-genome, gain or loss of mobile genetic elements, alterations in metabolic genes, or epigenetic modifications are responsible for the observed phenotypic variability.

In conclusion, our results suggest that reduced invasiveness, lower virulence, and superior carriage capacity are features of the serotype 3 phylogenetic clade II. Although an increase in pathogenic potential is unlikely to explain the recent epidemiological success of Pn3 clade II, mechanistic evidence establishing a direct phenotype-genotype link is required to further clarify current pneumococcal serotype 3 epidemiology dynamics.

## Supplementary Data

Supplementary materials are available at *The Journal of Infectious Diseases* online (http://jid.oxfordjournals.org/). Supplementary materials consist of data provided by the author that are published to benefit the reader. The posted materials are not copyedited. The contents of all supplementary data are the sole responsibility of the authors. Questions or messages regarding errors should be addressed to the author.

## Supplementary Material

jiae038_Supplementary_Data

## Notes

***Acknowledgments.*** We thank Urvi Rajyaguru, Lubomira Andrew, and Hal Jones for their help with sourcing and sequencing of pneumococcal isolates. We also thank Robert Donald, Julita Ramirez, and Ingrid Scully for critical reading of the manuscript; Nataliya Kushnir, Christina D’Arco, Laurent Chorro, Naveen Surendran, and Micah Dunlap for helpful discussions; and Paula Corts, Melanie Blanco, and Comparative Medicine colleagues for assistance with animal experiments.

***Financial support*.** This work was supported by the Division of Worldwide Research and Development, Pfizer, Inc.
