# Supplementary material for: Evidence of Reduced Virulence and Increased Colonization Among Pneumococcal Isolates of Serotype 3 Clade II Lineage in Mice
Source: J Infect Dis. 2024 Jan 29;230(1):e182–8. doi: 10.1093/infdis/jiae038 (PMC11272092; doi:10.1093/infdis/jiae038)
Supplement: jiae038_Supplementary_Data [file jiae038_supplementary_data.zip › SupplementaryTable1_FV.docx]

| Supplementary Table 1. Genomic, geographic, temporal, virulence, and carriage features of all tested isolates. | | | | | | | | | | | | |
| --- | --- | --- | --- | --- | --- | --- | --- | --- | --- | --- | --- | --- |
| Isolate ID | MLST | Clade | Country | Year | Isolation origin | LD_50_ | CFU/ml  day 14 | CFU/ml  day 21 | % colonized  mice day 14 | % colonized  mice day 21 | % euthanized mice day 14 | % euthanized mice day 21 |
| PFESP00646 | 180 | I-α | Canada | 2015 | Bronchial | 1.93×10^5^ | 1.26×10^2^ | 3.31×10^2^ | 60 | 60 | 0 | 0 |
| PFESP00695 | 180 | I-α | UK | 2015 | Sputum | 2.15×10^2^ | 9.06×10^1^ | <LOD | 20 | 10 | 60 | 70 |
| PFESP01039 | 180 | I-α | UK | 2016 | Nasal wash | 1.74×10^4^ | 1.16×10^1^ | <LOD | 20 | 0 | 10 | 10 |
| PFESP00169 | 180 | I-α | USA | 2008 | Sputum | >9.60×10^6^ | <LOD | <LOD | 0 | 20 | 0 | 0 |
| PFESP00670 | 505 | I-β | Spain | 2015 | Sputum | 8.21×10^4^ | 3.55×10^2^ | 3.04×10^2^ | 80 | 70 | 0 | 10 |
| PFESP00186 | 180 | I-β | Singapore | 2009 | Blood | 5.27×10^2^ | 2.60×10^1^ | <LOD | 40 | 30 | 30 | 20 |
| PFESP00105 | 180 | I-β | Brazil | 2007 | Blood | 7.23×10^5^ | 7.58×10^1^ | <LOD | 50 | 30 | 0 | 0 |
| PFESP00200 | 505 | I-β | China | 2010 | Blood | 9.10×10^4^ | 1.83×10^2^ | 3.50×10^1^ | 40 | 30 | 10 | 30 |
| PFESP00475 | 180 | II | USA | 2014 | Sputum | 3.86×10^6^ | 6.11×10^2^ | 2.01×10^2^ | 80 | 60 | 0 | 0 |
| PFESP00187 | 180 | II | Singapore | 2009 | Blood | 4.37×10^6^ | 9.64×10^2^ | 2.16×10^2^ | 80 | 60 | 10 | 0 |
| PFESP0068 | 180 | II | Germany | 2015 | Ears | >2.48×10^7^ | 2.83×10^2^ | 4.73×10^2^ | 60 | 80 | 0 | 0 |
| PFESP00136 | 180 | II | South Korea | 2007 | Blood | >1.10×10^7^ | 1.04×10^1^ | <LOD | 20 | 0 | 0 | 0 |
| PFESP00697 | 180 | II | USA | 2015 | Sputum | >9.00×10^6^ | 1.03×10^3^ | 7.01×10^1^ | 70 | 40 | 0 | 0 |
| PFESP01038 | 180 | II | UK | 2016 | Nasal wash | 1.63×10^6^ | 1.91×10^2^ | 6.98×10^1^ | 60 | 50 | 0 | 0 |
| MLST, multi-locus sequence type;  Clade, inferred from phylogenetic comparisons of Clonal Complex 180 (CC180) isolates;  Country, denotes country of isolation;  Year, denotes year of isolation;  Isolation origin, denotes the clinical or anatomical site of isolation;  LD50, lethal-dose 50, calculated using AAT Bioquest, Inc. (2021, May 14) Quest Graph™ Four Parameter Logistic (4PL) Curve Calculator.". Retrieved from <https://www.aatbio.com/tools/four-parameter-logistic-4pl-curve-regression-online-calculator>; isolates for which 4PL curves did not reach LD50, the highest challenge dose was given preceded by the sign “>”  CFU/ml, geometric mean of bacterial levels expressed as CFU/ml in nasal washes from a group of 10 challenged mice;  % colonized mice, calculated as the number of surviving mice with ≥10 CFU/ml in nasal washes / total number of challenged mice, expressed as percentage;  % euthanized mice, calculated the number of euthanized or dead mice at day 14 or 21 / total number of challenged mice, expressed as percentage. | | | | | | | | | | | | |
