# Supplementary material for: Evidence of Reduced Virulence and Increased Colonization Among Pneumococcal Isolates of Serotype 3 Clade II Lineage in Mice
Source: J Infect Dis. 2024 Jan 29;230(1):e182–8. doi: 10.1093/infdis/jiae038 (PMC11272092; doi:10.1093/infdis/jiae038)
Supplement: jiae038_Supplementary_Data [file jiae038_supplementary_data.zip › Supplementary_Figure_1_legend.docx]

**Supplementary Figure 1. ATCC 6303 serotype 3 strain shows a dose-dependent lethality and stable carriage up to 21 days post challenge.** Survival among challenged mice was evaluated for pneumococcal isolate ATCC 6303 and modeled using Four Parameter Logistic (4PL) dose-response (A). Each data point represents survival percentage from a group of 10 mice. Carriage capacity of the ATCC 6303 strain was evaluated by challenging groups of 10 mice with 104 CFU/animal (B). Each datapoint represents bacterial load (CFU/ml) from nasal washes at 14 and 21 days post challenge. Horizontal line represents the geometric mean average.
