# Supplementary figures and images for: Evidence of Reduced Virulence and Increased Colonization Among Pneumococcal Isolates of Serotype 3 Clade II Lineage in Mice
Source: J Infect Dis. 2024 Jan 29;230(1):e182–8. doi: 10.1093/infdis/jiae038 (PMC11272092; doi:10.1093/infdis/jiae038)

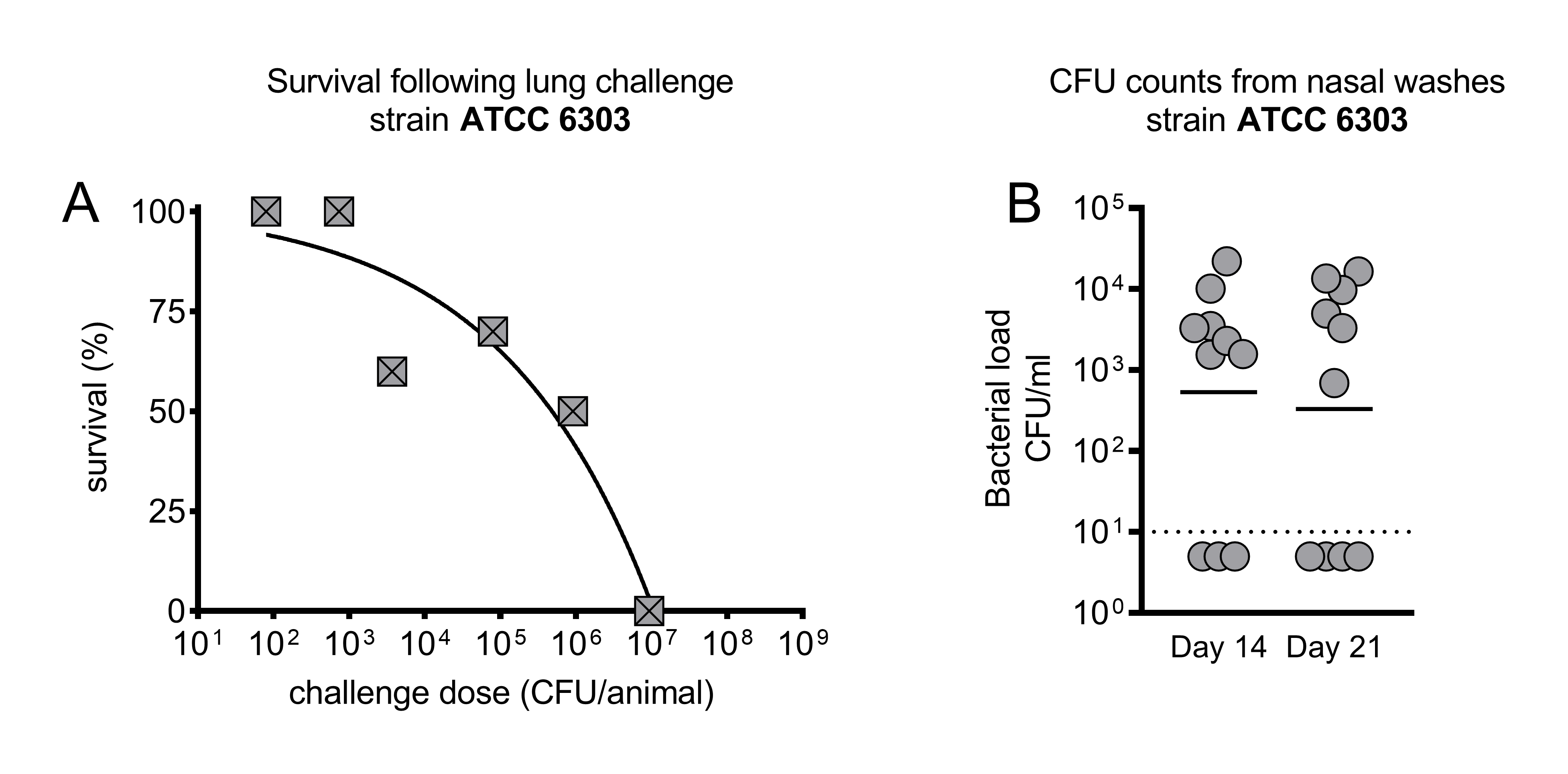

Supplement: jiae038_Supplementary_Data [file jiae038_supplementary_data.zip › SupplementaryFigure1.tif]
